# Practical Notes and Formulæ

**Published:** 1883-06-20

**Authors:** 


### PRACTICAL NOTES AND FORMULÆ.

**The Odor of Iodoform.**—Having tried nearly all the devices that have been suggested for mitigating or disguising the odor of iodoform, and found them all of little or no avail, we have lately come nearer to the object by using oil of eucalyptus, according to the following formula :

℞ Pulv. iodoform.................................. ℥ ss,

Ol. eucalypti................................. f. ʒ ss,

Vaselin........................................ ℥ iv,

M., fiat unguentum.

We do not remember to have seen any account of the oil having been used for this purpose by others. The ointment thus prepared is not without odor, but the odor is not that of iodoform.—*N. Y. Med. Jour. and Obst. Review.*

**A Little Girl,** ten years of age, was afflicted with tuberculosis of the lungs. She was pale, emaciated, and harassed by a cough. The physical signs were those of the second stage of the disease. Dr. Bruen prescribed :

℞ Olei morrhuæ.................................. fl. ℥ i,

Syr. calcii lactophosphatis..................... fl. ʒ ij

Syr. ferri iodidi................................ fl. ʒ j,

Liquor calcis.................................. fl. ℥ ij.

M. Sig. A teaspoonful three times a day after meals.

As an embrocation, equal parts of cod-liver oil and soap liniment were ordered. The patient was to wear warm flannels and take outdoor exercise. For the cough :

℞ Acid. sulphuric dil............................ *m* xvj,

Tr. opii deodorat............................. *m* viij,

Syr. pruni virgin.............................. fl. ℥ j,

Aquæ, q. s. ad................................. fl. ℥ ij.

M. Sig. A teaspoonful or two every two or three hours.—*Ibid.*

**Aphthous Sore Mouth in Infants.**—Prof. Wallace, in College and Clinical Record, recommends the following :

R Sodii sulphitis................................ gr. xxx,

Glycerinæ..............................}

Aquæ.....................................} aa ℥ ss.

M. To be used on a swab every two hours.

Scrupulous cleanliness is required when a nursing bottle is used. The rubber nipple should be turned inside out after each using, washed clean, and kept in a solution of baking soda until again needed. It is better to have two nipples, and to use them alternately. Milk must not be allowed to stand in the bottle till it grows sour.—*Ame. Med. Jour.*

**Hop Cordial.**—The following is commended as a palatable preparation, not inferior to any of the vaunted "Hop Bitters :"

| | |
|---|---|
| Hops | 2 oz, |
| Dandelion | 2 oz, |
| Gentian | 2 oz, |
| Chamomiles | 2 oz, |
| Stillingia | 2 oz, |
| Orange peel | 2 oz, |
| Alcohol | 77 fluid oz, |
| Water | 77 fluid oz, |
| Simple syrup | 12 fluid oz. |

Exhaust the drugs with the alcohol and water, and add the simple syrup.—*Pop. Sci. News.*

**Remedy for Baldness.**—The following formula is highly commended for baldness—

| | |
|---|---|
| Tinct. capsici annui | ℥i, |
| Glycerini | ℥iij, |
| Ol. bergamot | ʒi. |

To be rubbed into the skin of the head.—*Ibid.*

**Cracked Nipples.**—

| | |
|---|---|
| Bismuth sub nit | 2 drachms, |
| Vaselini | 1 ounce. |

M. Sig. Apply to the nipple each time after the child has nursed, and cover with a soft cloth. The ointment should be washed off before applying the child again to the breast. This remedy may not be anything new to many of your readers, but it may help some who have never tried it. With me the results have been perfectly satisfactory.—*Peoria Med. Monthly.*

Case —. Male 48 years old; chronic gastritis with hyperæmia of liver and cirrhosis in first stage, with all the accompanying symptoms. Caused by alcoholism. Prescribed :

| | |
|---|---|
| ℞ Fl. ext. cascara sagrada | gtt. x, |
| Fl. ext. wood betony | gtt. xxv, |
| Tinc. card. co. | aa ʒ ss. |
| Simple syrup | |

M. Sig. Three times a day, before meals.
Patient entirely cured in two months.—*Thera. Gaz.*

**Emmenagogue.**—

| | |
|---|---|
| ℞ Fl. ext. ergot | |
| Fl. ext. gossypii | aa ℥j. |
| Fl. ext. black cohosh | |

M. Sig. Half a teaspoonful every three hours, and using hot fomentations of hops on the bowels.—*Ibid.*

**To Kill Rats and Mice.**—A French writer gives the following recipes for this purpose, which he has tested and found good:

(1.) 120 grams (say 12 parts by weight) of crumbs of bread, 60 grams (6 parts) of butter, 50 grams (3 parts) crystalized nitrate of mercury, all well mixed together in an impalpable paste; lay some of the mixture on pieces of glass in the house where the mice are.

(2.) 250 grams (5 parts) quicklime (not slaked) in powder, 50 grams (1 part) sugar in powder, 150 grams (3 parts) flour of any kind (oat, wheat or rye); mix well together, put some of the mixture on a little plate, and place near it a second plate with water. Mice, after eating some of the mixture feel thirsty and drink the water. The lime, being quick, gets slaked in their stomachs, and kills them in a few minutes.—*Ex.*

**Expectorant Mixtures in Bronchitis.**—

| | | |
|---|---|---|
| ℞ | Carb. of ammon | ʒ i, |
| | Fl. ext. of squills | aa ʒ ij, |
| | Fl. ext. of senega | |
| | Paregoric | ℥ iss, |
| | Water | ℥ i, |
| | Syr. of tolu | ℥ v. |

M. Dose from three to four teaspoonfuls as may be required.

---

| | | |
|---|---|---|
| ℞ | Iodide of potassium | ʒ iiss, |
| | Syrup of tolu | aa ℥ ii, |
| | Glycerine | |
| | Sulph. morphia | gr. ij. |

M. Dose, a tablespoonful once in four or six hours.—*Mich. Med. News.*

**Laxative for Gouty and Rheumatic Subjects:**

| | | |
|---|---|---|
| ℞ | Pulv. rhei | aa gr. xx, |
| | Sodæ bicarbonat | |
| | Infus. rhei | ℥ i. |

Make a draught to be taken early in the morning with two or three tablespoonfuls of water twice or thrice a week.—*Ibid.*

**A Specific for Singultus.**—This very common affection, of infants and children especially, has a specific remedy, at least one which I have never known to fail. Moisten granulated sugar with good cider-vinegar; give to an infant from a few grains to a teaspoonful. The effect is almost instantaneous, and the dose seldom needs to be repeated. I have used it for all ages, from infants a few months old, to those on the down-hill side of life. Try it.—*Henry Tucker, M. D., Brattleboro, Vt.*

**Nothing** is better for whitening garments, particularly those that have become yellow from being laid aside for several months, than a teaspoonful of borax dissolved in the rinsing water.—*Ex.*

